# Combination of CTLA-4 blockade with MUC1 mRNA nanovaccine induces enhanced anti-tumor CTL activity by modulating tumor microenvironment of triple negative breast cancer

**DOI:** 10.1016/j.tranon.2021.101298

**Published:** 2021-12-04

**Authors:** Xuan Lin, Hedan Chen, Ying Xie, Xue Zhou, Yun Wang, Jing Zhou, Shiqi Long, Zuquan Hu, Shichao Zhang, Wei Qiu, Zhu Zeng, Lina Liu

**Affiliations:** a Key Laboratory of Biological and Medical Engineering/Immune Cells and Antibody Engineering Research Center of Guizhou Province/Engineering Research Center of Medical Biotechnology, School of Biology and Engineering, Guizhou Medical University, Guiyang, Guizhou 550025, China; b Key Laboratory of Environmental Pollution Monitoring and Disease Control, Ministry of Education, Guizhou Medical University, Guiyang, Guizhou 550025, China; c School of Basic Medical Science, Guizhou Medical University, Guiyang, Guizhou 550025, China

**Keywords:** MUC1 mRNA nanovaccine, CTLA-4 blockade, Combined therapy, Tumor microenvironment, Triple negative breast cancer

## Abstract

The immunosuppressive tumor microenvironment (TME) is the main reason for the failure of many immunotherapies that directly stimulate anti-tumor immune response. Anti-CTLA-4 antibody may reduce effector regulatory T (Treg) cell numbers and their suppressive activity in the TME. We have previously reported that combination of anti-CTLA-4 antibody with MUC1 mRNA nanovaccine may mutually enhance each single treatment. But the enhancement mechanism of therapeutic efficacy of MUC1 mRNA nanovaccine plus anti-CTLA-4 monoclonal antibody (mAb) is unknown. In this study, anti-tumor CTL activity induced by combination of CTLA-4 Blockade with MUC1 mRNA nanovaccine and immunosuppressive factors in the TME of triple negative breast cancer were investigated. The results demonstrated that combined therapy with nanovaccine and anti-CTLA-4 mAb could induce stronger anti-tumor CTL response than each monotherapy, result in significantly decreased numbers of myeloid-derived suppressor cells (MDSC), Treg cells, tumor-associated fibroblasts (TAFs) and tumor vasculature in the TME, downregulated levels of interleukin-6, tumor necrosis factor-α and transforming growth factor-β, and significantly upregulated levels of IFN-γ and interleukin-12 as well as increased number of CD8^+^ T cell, and appear more effective than either nanovaccine or anti-CTLA-4 mAb alone at increasing level of apoptosis in tumor cells. In addition, combination immunotherapy could significantly downregulated the signal transducer and activator of transcription 3 (STAT3) signal pathway. Therefore, it can be concluded that combination of CTLA-4 blockade with MUC1 mRNA nanovaccine enhances anti-tumor cytotoxic T-lymphocyte activity by reducing immunosuppressive TME and inhibiting tumor-promoting STAT3 signaling pathway.

## Highlights

• Combined therapy with MUC1 mRNA nanovaccine and anti-CTLA-4 mAb could reduce immunosuppressive TME, increase the infiltration of CD8^+^ T cells into tumor sites, and enhance anti-tumor cytotoxic T-lymphocyte activity when compared with each monotherapy.
• Combination treatment with anti-CTLA-4 mAb and MUC1 mRNA nanovaccine could appear more effective than either nanovaccine or anti-CTLA-4 mAb alone at increasing level of apoptosis in tumor cells.
• Combination immunotherapy could significantly downregulated the signal transducer and activator of transcription 3 (STAT3) signal pathway.

## Introduction

Triple-negative breast cancer (TNBC) is a subtype of breast cancer that does not express estrogen receptor (ER), progesterone receptor (PR) and human epidermal growth factor receptor 2 (HER2) [1]. Because there are no well-defined molecular targets, the treatment of patients with TNBC remains a great clinical challenge [2]. Immunotherapy is emerging as a promising treatment approach for TNBC [3]. The Cancer-Immunity Cycle contains seven major steps, starting with the release of cancer cell antigens and ending with the killing of cancer cells by T cells [4]. If the Cancer-Immunity Cycle is blocked at one or more of the seven steps, immune escape is most likely to occur. Recent evidence indicates that despite the presence of cytotoxic T cells in the tumor microenvironment (TME) of TNBC, the tumor still can progress and metastasize, suggesting immune evasion [2, 3]. Immunosuppression mechanisms include the presence of inhibitory cytokines, immune evasion molecules and inhibitory enzymes, induction of tolerogenic cell death and existence of dense extracellular matrix in the TME [5]. Immunosuppressive TME avoids immune recognition and elimination [6]. The goal of cancer immunotherapy is to initiate or re-implement the self-sustaining Cancer-Immunity Cycle for elimination of cancer by T cells [4]. Cancer vaccines are designed to increase cancer antigen presentation in DCs and improve antitumor immune response [7]. TME modulation is to restore a natural antitumor immune capacity and enhance the ability to kill targeted cancer cells [8].

Cancer vaccines and checkpoint blocking antibodies are currently explored as potential treatment strategies for breast cancer [9]. Vaccine is a form of active immunotherapy to induce specific immune response to tumor antigens. Checkpoint blocking antibodies may inhibit immune suppression by targeting key pathways mediated by immune checkpoint molecules, such as cytotoxic T-lymphocyte-associated antigen 4, programmed death 1 (PD-1), and programmed death ligand 1 (PD-L1) [9]. After T cell activation, CTLA-4 is expressed on the surface of activated T cells. As a homolog of CD28, CTLA-4 has a much higher affinity for binding B7 molecules and can directly compete with CD28 to eventually attenuate T cell activation [10]. CD28 signals drive critical T cell effector functions, contribute to enhanced cytokine production, influence T cell migration [11]. Therefore, effective control of CD28 co-stimulation is absolutely necessary and can be achieved by targeting CTLA-4 pathway. Regulatory T cells (Treg cells) also exhibit constitutive expression of CTLA-4 [12]. CTLA-4 plays an important role in regulating immunological self-tolerance [12]. Anti-CTLA-4 antibody may kill tumor-infiltrating effector Treg cells or decrease their suppressive activity [13]. Tremelimumab, a monoclonal antibody specific for CTLA-4, is currently being investigated in patients with TNBC [1]. The efficacy of anti-CTLA-4 monotherapy is limited or ineffective, suggesting the need for combinations with other therapeutic strategies.

Combinations within a step and across steps of Cancer-Immunity Cycle may be clinically beneficial [14]. Combinations of immune checkpoint blockade with targeting other immune checkpoints is moving forward in TNBC [15]. Preclinical and clinical data supports the use of cancer vaccines with anti-CTLA-4 antibody [16]. Tumor-targeted radiotherapy (RT) is used to generate an in situ tumor vaccine [17]. Combination of tumor-targeted RT with anti-CTLA-4 can induce effective immune responses to poorly immunogenic tumors 4T1 mouse TNBC [17, 18]. In some clinical trials, tumor vaccine is used with CTLA-4 blockade for TNBC treatment [15]. Previously, we have reported that combination immunotherapy of MUC1 mRNA nanovaccine and CTLA-4 blockade can effectively inhibit growth of triple negative breast cancer and significantly increase the number of tumor-infiltrating CD8^+^T cells compared to the vaccine or anti-CTLA-4 monoclonal antibody alone [19]. Higher tumor-infiltrating lymphocytes in TNBC are associated with improved overall survival (OS) and higher response rate [15]. However, the expression of immunosuppressive molecules and the infiltration of inhibitory immune cells into the tumor microenvironment will significantly inhibit cytotoxic T lymphocyte (CTL)-mediated tumor killing effect [20]. We hypothesized that the combination treatment of anti-CTLA-4 monoclonal antibody and MUC1 mRNA nanovaccine may reduce immunosuppressive TEM, normalize tumor vasculature and inhibit tumor-promoting signaling pathways to achieve increased anti-tumor CTL activity and enhanced inhibition of TNBC growth.

## Materials and methods

### Materials

1,2-Distearoryl-sn‑glycero-3-phosphoethanolamine-N-(methoxy[*polyethyleneglycol*-2000]) ammonium salt (DSPE-PEG) was purchased from NOF Corporation (Tokyo, Japan). DSPE-PEG-mannose was obtained from Xi'an Ruixi Biological Technology Co., Ltd (Shanxi, China). Dioleoylphosphatydic acid (DOPA) and 1,2-dioleoyl-3-trimethylammonium-propane chloride salt (DOTAP) were sourced from Avanti Polar Lipids (Alabaster, AL, USA). Cholesterol, cyclohexane, and IGEPAL-CO-520 were purchased from Sigma-Aldrich (St. Louis, MO, USA). All other chemicals were purchased from Sigma-Aldrich if not specifically mentioned. Nucleotides (ATP and guanosine triphosphate [GTP]) were purchased from Thermo Fisher Scientific (Waltham, MA, USA). Modified nucleotides (5-methylcytidine-5′-triphosphate and pseudouridine-5′-triphosphate) were obtained from Trilink Biotechnologies (San Diego, CA, USA). 3′-O-Me-m^7^G (5′) ppp (5′) G RNA cap structure analog was purchased from NEB (Ipswich, MA, USA).

### Cell line and mice

The TNBC 4T1 cell line, which is derived from a spontaneous mammary carcinoma in a BALB/c mouse, was obtained from ATCC Shanghai Cell Bank, Chinese Academy of Sciences, and cultured in a Roswell Park Memorial Institute (RPMI) 1640 medium, supplemented with 10% fetal bovine serum (GIBCO), 100 U/mL penicillin, and 100 μg/mL streptomycin at 37℃ in a humidified 5% CO_2_ atmosphere. 6- to 8-week old female BALB/c mice were obtained from Chongqing Tengxin Biotech Co., Ltd (Chongqing, China). All work performed on animals was in accordance with and approved by Guizhou Medical University's Institutional Animal Care and Ethics Committee.

### Antibodies

Anti-CTLA-4 (9D9) and mouse immunoglobulin G2b (IgG2b) isotype control used in vivo were purchased from BioXCell (West Lebanon, NH, USA). Primary antibodies used for western blot analysis and immunofluorescence staining included anti-STAT3 monoclonal antibody, anti-phospho-STAT3 monoclonal antibody, anti-α-SMA monoclonal antibody (Cell Signaling Technology, Danvers, MA, USA), anti-mouse CD8α polyclonal antibody, anti-CD31 polyclonal antibody (Servicebio, Wuhan, China) and anti-GAPDH monoclonal antibody (Zsbio, Beijing, China). Secondary antibody used for western blot analysis and immunofluorescence staining included goat anti-mouse IgG-horseradish peroxidase, goat anti-rabbit IgG-horseradish peroxidase (Abmart, Shanghai, China) and Alexa Fluor 647 conjugated anti-rabbit (Cell Signaling Technology). FITC-conjugated anti-mouse CD8α, PE-conjugated anti-mouse FOXP3, PE-conjugated anti-mouse Gr, and FITC-conjugated anti-mouse CD4 were obtained from Thermo Fisher Scientific (Waltham, MA, USA). FITC-conjugated anti-mouse CD11b, PE-conjugated anti-mouse CD3 and Fc block were purchased from BD Biosciences (San Diego, CA, USA). These primary antibodies were used for flow cytometry assay.

### MUC1 mRNA nanovaccine

Lipid/calcium/phosphate (LCP) NPs was used for the delivery of MUC1 mRNA to dendritic cells (DCs) in lymph node and LCP-based mRNA vaccine expressing complete MUC1 protein of mouse was prepared as previously described [19]. In brief, the calcium solution (600 μL 2.5 M CaCl_2_ and 50 μg MUC1 mRNA) and phosphate solution (600 μL 12.5 mM Na_2_HPO_4_) were separately added to the two stirring cyclohexane/Igepal CO-520 (71:29, V/V) oil phases (20 mL/bottle), and then the calcium phase and phosphate phase were separately stirred for 5 min at room temperature. Subsequently, the two oil phases were mixed and stirred for 20 min at room temperature. After the addition of 400 mL 20 mM DOPA, the microemulsion was stirred further for 15 min. In order to precipitate the calcium phosphate cores, 40 mL ethanol was added. Then, the calcium phosphate cores were collected using centrifugation at 10,000×*g* for 20 min and washed with ethanol to remove the remaining cyclohexane and Igepal. The pellets were dissolved in 2 mL chloroform. The final particles were prepared by adding 140 μL 20 mM DOTAP, 140 μL 20 mM cholesterol, 100 μL 20 mM DSPE-PEG-2000, and 80 μL 5 mM DSPE-PEG-mannose to the LCP core. After chloroform evaporation, the LCP-based MUC1 mRNA vaccine was resuspended in 250 μL of 5% glucose solution and sonicated before administration. The LCP cores and LCP NPs were observed by transmission electron microscopy (TEM).

### In vivo antitumor effect

6- to 8-week old female BALB/c mice were inoculated with 1 × 10^5^ 4T1 tumor cells in the mammary fat pad. Mice were randomly divided into 6 groups (*n* = 7–10 per group) with different treatments as follows: PBS group (PBS), mRNA group (naked MUC1 mRNA), LCP group (empty LCP NPs), LCP-mRNA group (LCP NPs loaded with MUC1 mRNA), CTLA-4 group (anti-CTLA-4 monoclonal antibody), Combination group (LCP-based MUC1 mRNA vaccine plus anti-CTLA-4 monoclonal antibody). PBS group (100μL PBS per dose), mRNA group (10 μg mRNA per dose), LCP group (100μL LCP per dose), LCP-mRNA group (10 μg mRNA per dose), and Combination group (10 μg mRNA plus 100 μg mAb/mouse) were s.c. injected on day 6 and 13 after tumor inoculation. For CTLA-4 group (100 μg mAb per dose) and combination group, mAb was i.p. injected on day 3, 6, 9 and 12 post-tumor injection. Tumor size was monitored. At the endpoint of tumor inhibition experiment on day 19, mice were euthanized. Tumors were harvested, weighed and tested.

### Flow cytometry assay

Tumor-infiltrating immune cells were analyzed by flow cytometry. Single cells were collected from fresh tumor tissues after digestion with collagenase A and DNase I at 37 °C for 40 min. For the staining of surface markers, cells were pre-incubated with Fc block and stained with different fluorescein-conjugated antibodies. For intracellular marker staining, the cells were fixed and permeabilized using the Fixation/permeabilization buffer (BD Biosciences), and then stained with fluorescent antibodies.

### Real-time qPCR assay

Total RNAs were extracted from the tumor tissues by using TRIzol Reagent (Invitrogen, Carlsbad, CA, USA) and reverse transcribed to cDNA using SuperScript III First-Strand Synthesis System (Invitrogen). RT-qPCR was performed using the TB Green® Premix Ex Taq II (Tli RNaseH Plus), ROX plus (Takara, Beijing, China) according to the manufacturer's instructions. β-actin was used as an internal control. The mouse-specific primers for RT-qPCR were listed in Table 1. Reactions were conducted using the StepOnePlus Real-Time PCR System.

**Table 1:** Mouse-specific primers for RT-qPCR.

Primer	Sequence (5′−3′)
TNF-α	F: ACTGGCAGAAGAGGCACTCC R: GCCACAAGCAGGAATGAGAA
TGF-β	F: ACTTGCACCACCTTGGACTTC R: GGTCATCACCGTTGGCTCA
IL-12	F: AAGACATCACACGGGACCAAACCA R: CGCAGAGTCTCGCCATTATGATTC
IL-6	F: CGGAGAGGAGACTTCACAGAG R: ATTTCCACGATTTCCCAGAG
IFN-γ	F: GACAATCAGGCCATCAGCAACAAC R: GAGGCTGGATTCCGGCAACAG
β-actin	F: GTGCTATGTTGCTCTAGACTTCG R: ATGCCACAGGATTCCATACC

### Immunofluorescence staining

Paraffin-embedded tissue sections were blocked in 5% bovine serum albumin for 1hr at room temperature after deparaffinization and antigen retrieval. Primary antibodies were incubated overnight at 4 °C, followed by incubation with fluorescent secondary antibody at room temperature for 1hr. The nuclei were counterstained with DAPI (Solarbio, Beijing, China). Images were acquired using fluorescence microscopy (Nikon, Tokyo, Japan) and analyzed by ImageJ software.

### In vivo CTL assay

The CTL assay was performed according to previously published protocol with slight modifications [19]. Female BALB/c mice were immunized with PBS, LCP-based MUC1 mRNA vaccine, anti-CTLA-4 mAb, MUC1 mRNA vaccine plus anti-CTLA-4 mAb, and control mAb, respectively. After seven days, the immunized mice were injected intravenously with splenocytes from naïve BALB/c mice, half of splenocytes were pulsed by lysates of 4T1 cells transfected with MUC1 mRNA (2 μg/μL) with CFSE^high^ (4 μM) and the other half were pulsed by CT26 cell lysates (2 μg/μL) with CFSE^low^ (0.4 μM). After 18 hr, splenocytes were separated from the spleens of treated mice and analyzed by flow cytometry. MUC1-specific lysis percentage in vivo was calculated according to the equation below.

%MUC1-specific lysis = (CT26 cell lysates × *x* - transfected cell lysates)/(CT26 cell lysates × *x*) × 100%

### ELISPOT assay

Female BALB/c mice were immunized with PBS, LCP-based MUC1 mRNA vaccine, anti-CTLA-4 mAb, MUC1 mRNA vaccine plus anti-CTLA-4 mAb, and control mAb, respectively. After seven days, spleens were sterilely harvested and separated into single cells. 2 μg/μL 4T1 cell lysates transfected with MUC1 mRNA or CT26 cell lysates were cocultured with the cells (2 × 10^6^ per well) seeded in a capture antibody-coated 96-well plate at 37 °C for 18 hr. IFN-γ production was measured using Mouse IFN-γ ELISPOT Kit (BD Biosciences) according to the manufacturer's instructions. Red dot signals were manually enumerated under the microscope.

### Western blot analysis

The total proteins were extracted using One Step Animal Tissue Active Protein Extraction Kit (Sangon Biotech, Shanghai, China) from tumor tissues. The total protein concentrations were measured using BCA Protein Assay Kit (Sangon Biotech). After the same amount of extracted proteins were separated by SDS-PAGE and transferred onto polyvinylidene fluoride membranes (Merck Millipore), the protein-loaded membranes were incubated with anti-phospho-STAT3, anti-STAT3, and anti-GAPDH antibody, respectively. After incubation with horseradish peroxidase conjugated secondary antibody, the membranes were monitored using the ECL luminescence reagent (Sangon Biotech). ImageJ software was used to calculate the band densities of proteins.

### TUNEL assay

Apoptotic tumor cells in the paraffin-embedded tumor tissues were characterized by using TUNEL assay kit (Promega, Madison, WI). The TUNEL assay was performed according to TUNEL System instruction. The nuclei were double stained with Propidium iodide (PI) (Macklin, Shanghai, China). FV1000 laser-scanning confocal microscopy (Olympus, Tokyo, Japan) was used for observation. Three randomly selected fields were quantitatively analyzed using ImageJ software.

### Statistical analysis

Data are shown as mean ± SEM. GraphPad Prism software was used to perform the statistical analyses using a two-tailed t-test, when comparing two groups, and one-way ANOVA when comparing more than two groups. Differences were considered statistically significant for *p < 0.05, **p < 0.01, ***p < 0.001, and non-significant for *p* > 0.05.

## Results

### Expression of MUC1 mRNA in vitro and characterization of MUC1 mRNA-loaded LCP NP

LCP NP that consist of a calcium phosphate core and an asymmetrical lipid bilayer were first developed for the purpose of siRNA delivery [21]. Twenty-four hours after transfection with mRNA encoding EGFPs loaded LCP NP in vitro, 68% of the DCs expressed EGFP [22]. Our previous experiment demonstrated that the encapsulated MUC1 mRNA into LCP NP could be successfully expressed in the lymph nodes on day 7 after vaccination, and the encapsulation efficiency was about 50% [19]. MUC1 mRNA nanovaccine targeting DCs in the lymph node was prepared as previously described [19]. The encapsulated MUC1 mRNA was transcribed and modified in vitro (Supplementary Fig. S1A). Western blot analysis indicated that the in vitro transcriptionally modified MUC1 mRNA could be transiently expressed in mammalian 4T1 cells (Supplementary Fig. S1B). Then, the MUC1 mRNA was encapsulated into LCP. Transmission electron microscopy (TEM) pictures showed that LCP core (Supplementary Fig. S2A) and LCP NP (Supplementary Fig. S2B) were about 15 nm and 50 nm in diameter, respectively.

### Combination treatment with anti-CTLA-4 mAb and MUC1 mRNA nanovaccine improved antitumor effect

Tumor vaccine may release tumor-associated antigens, which may lead to recruit and activate Treg cells in tumor tissues, consequently hindering ensuing antigen specific anti-tumor response. Therefore, in order to activate the effector T cells strongly, it is necessary to deplete Treg cells or reduce their inhibitory activity before vaccine immunization [13]. Anti-CTLA-4 antibody may reduce effector Treg cell numbers or reduce their suppressive activity [13]. Combination of Treg cell attenuation by reducing its suppressive activity in tumor tissues with tumor-specific effector T cell activation by cancer vaccine may mutually enhance each single treatment [13]. Thus, MUC1 mRNA nanovaccine was combined with anti-CTLA-4 mAb for the enhancement of immunity against TNBC. Anti-CTLA-4 mAb was injected on day 3, 6, 9 and 12 prior to MUC1 mRNA nanovaccine immunization on day 6 and 13. It could be seen from Figure S3A and Figure S3B that both the MUC1 nanovaccine group and anti-CTLA-4 mAb group could significantly inhibit tumor growth compared to the PBS group. Compared with either the MUC1 nanovaccine group or the anti-CTLA-4 mAb group, the combo group exhibited a superior antitumor effect. At the endpoint of tumor inhibition experiment, the tumor weight of nanovaccine group and anti-CTLA-4 mAb group was significantly lower than that of PBS group, and the tumor weight of the combo group was significantly lower than that of each single treatment group. The results suggested that combination of anti-CTLA-4 mAb with MUC1 mRNA nanovaccine, the injection of anti-CTLA-4 mAb prior to vaccination, could improve each individual therapy against TNBC.

### Changes of tumor-infiltrating immune cells and cytokines in the TME

**Fig. 1:**
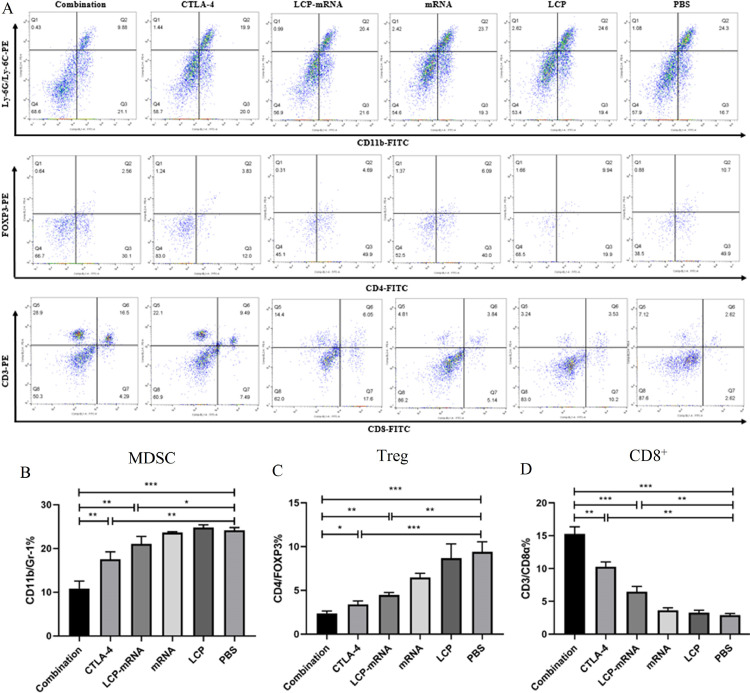
**Change of tumor-infiltrating immune cells in the TME.** (A) 4T1 breast cancer-bearing mice were randomly divided into 6 groups and treated with PBS, empty LCP, naked MUC1 mRNA, LCP-based MUC1 mRNA vaccine, anti-CTLA-4 monoclonal antibody, and LCP-based MUC1 mRNA vaccine plus anti-CTLA-4 monoclonal antibody, respectively. At the end of treatment (day 19), tumor tissues were harvested. Tumor single-cell suspensions were stained and analyzed using flow cytometry. (B, C, D) The percentages of MDSC, Treg and CD8^+^ T cells in tumors were statistically analyzed. *p < 0.05, **p < 0.01, ***p < 0.001; *n* = 3.

The TEM plays the crucial role in cancer development and progression, and has also been shown to participate in the regulation of tumor immune escape [23, 24]. Tumor cells, immunosuppressive immune cells, fibroblasts, neovasculature and secreted inhibitory cytokines in the TME are involved in hampering anti-tumor immune responses [5, 6, 25]. Combined therapy to reduce the immunosuppressive TME can be a promising strategy [5]. Depletion of Tregs and myeloid-derived suppressor cells (MDSCs) will enhance anti-tumor immune responses [25]. Fig. 1 showed that the percentages of Treg and MDSC in the combined treatment group, anti-CTLA-4 mAb group and nanovaccine group were significantly lower than that in the PBS group, whereas the percentage of CD8^+^ T cells in those group were much higher than that in the PBS group. Compared with the anti-CTLA-4 mAb group and nanovaccine group, the combo group exhibited an obvious decrease in Treg and MDSC, and a significant increase in the number of CD8^+^ T cells, indicating the ability to remodel the immunosuppressive TME by combined therapy.

**Fig. 2:**
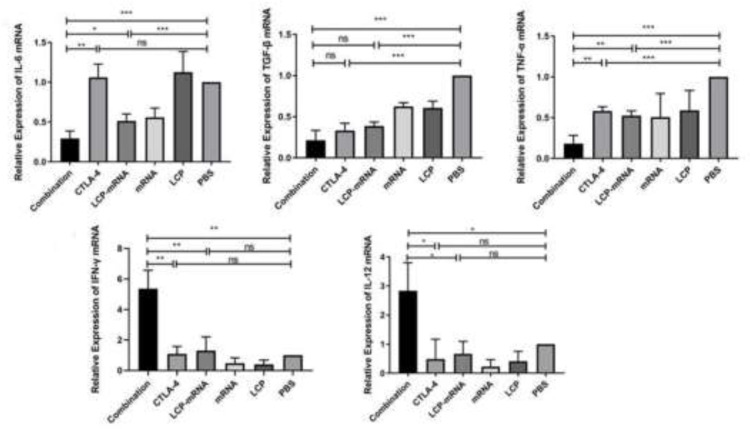
**Detection of cytokines in the TME using RT-qPCR.** 4T1 breast cancer-bearing mice were randomly divided into 6 groups. At the end of different treatments (day 19), tumor tissues were harvested. Total RNAs were extracted from the tumor tissues followed by cDNA synthesis. Then, qPCR was used for gene expression analysis of IL-6, TGF-β, TNF-α, IL-12 and IFN-γ. *p < 0.05, **p < 0.01, ***p < 0.001, ns: no significant difference; *n* = 3.

Interleukin (IL)−6 and transforming growth factor-β (TGF-β) are considered as important contributors to immune suppression in the TME and highly produced by TNBC cells [26]. IFN-γ and tumor necrosis factor-α (TNF-α) are often regarded as the cytokines secreted by cytotoxic T cells, which contribute to T-cell killing and fight against tumor progression [6]. It has also been reported that TNF-α secreted by macrophages induces TNBC immunosuppression via NF-κB signaling [2, 27]. Interleukin (IL)−12 can enhance the effect of active immunotherapy [25]. Therefore, these cytokines in tumor tissues were examined by RT-qPCR. As shown in Fig. 2, in the combo group and nanovaccine group, IL-6, TGF-β and TNF-α were significantly decreased, whereas in the anti-CTLA-4 mAb group, TGF-β and TNF-α were obviously decreased, but IL-6 didn't reduce when compared with the PBS group. Only in the combo group, IL-12 and IFN-γ greatly increased compared with the PBS group. IL-6, TGF-β and TNF-α were largely decreased in the combo group, while IL-12 and IFN-γ were significantly increased compared with the nanovaccine group and anti-CTLA-4 mAb group, indicating the decrease of immunosuppressive cytokines in the TME and the enhancement of cytotoxic T cell-mediated tumor-specific killing effect.

### Changes of tumor-associated fibroblast, tumor vasculature and CD8^+^T cells in TME

**Fig. 3:**
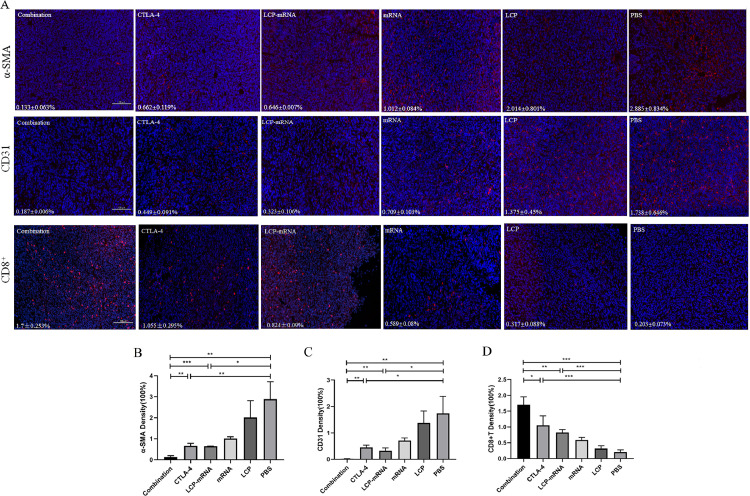
**Changes of tumor-associated fibroblast, tumor vasculature and CD8^+^T cells in TME.** (A) 4T1 breast cancer-bearing mice were treated with PBS, empty LCP, naked mRNA, LCP-based mRNA vaccine, anti-CTLA-4 monoclonal antibody, and both mRNA vaccine and anti-CTLA-4 monoclonal antibody, respectively. On day 19, tumor tissues were harvested for immunofluorescence staining of α-SMA, CD31 and CD8. DAPI stained the cell nuclei. Scale bar, 100 μm. (B) Three randomly selected fields were quantitatively analyzed using ImageJ software. *p < 0.05, **p < 0.01, ***p < 0.001.

Tumor-associated fibroblasts (TAFs) form a predominant stromal cellular component of the tumor microenvironment, which may promote tumor progression and limit effector T-cell activity [28, 29]. Depletion of TAFs may enhance the infiltration of effective immune cells into the tumor [29]. The tumor vasculature is a key component of the microenvironment that can block access to the tumor and influence treatment response. Vascular normalization may improve the delivery of immunotherapies and the trafficking of immune effector cells [23]. Thus, TAFs, tumor vasculature and CD8^+^ T cells in tumor tissues were detected by immunofluorescence staining after combined therapy. As shown in Fig. 3, the density of α-smooth muscle actin (α-SMA) (a marker of TAFs) and CD31 (a marker for tumor vasculature) in the nanovaccine and anti-CTLA-4 mAb groups was lower than that of the PBS group, whereas the density of CD8 (a marker for CD8^+^ T cells) was higher than that of the PBS group. The combo group exhibited the lowest density of α-SMA and CD31 and the highest density of CD8, suggesting that the decrease of TAFs and normalization of tumor vasculature in the TME could enhance the infiltration of CD8^+^ T cells into tumor sites.

### Combined therapy with anti-CTLA-4 mAb and MUC1 mRNA nanovaccine enhance the vaccine-inducing cytotoxic T-lymphocyte activity

**Fig. 4:**
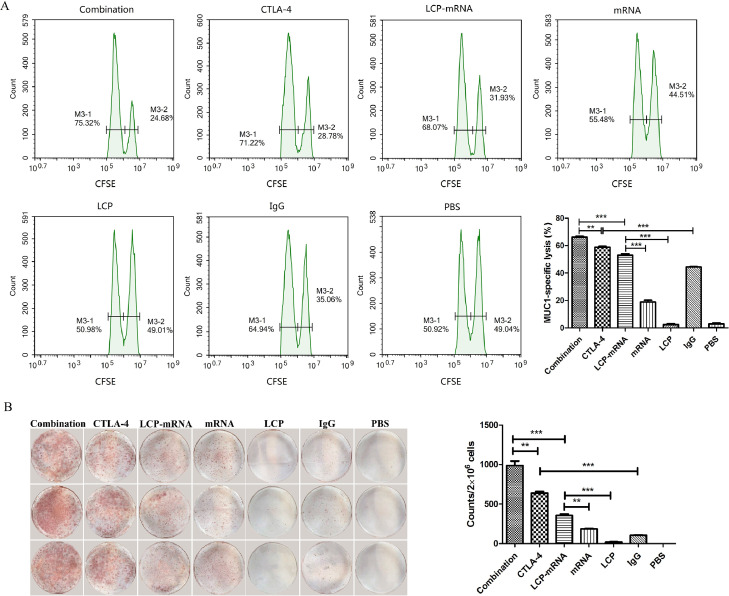
**Combined therapy with anti-CTLA-4 mAb and mRNA nanovaccine enhance the vaccine-inducing cytotoxic T-lymphocyte activity.** (A) *In vivo* CTL response after various treatments. One representative graph from each group is shown. *n* = 4. (B) IFN-γ production detected by ELISPOT assay after vaccination. *n* = 3. **p < 0.01, ***p < 0.001.

An *in vivo* CTL assay was performed to assess the ability to activate CTLs and CTL-mediated lysis. Antigen-specific lysis was analyzed using flow cytometry. Actually, after T cell activation, CTLA-4 is expressed on the surface of activated T cells, and Treg cells also constitutively express CTLA-4, which would impair activation and expansion of anti-tumor T cells [30]. Combined Therapy with vaccine and anti-CTLA-4 mAb enhanced CD8^+^ T cell infiltration of tumors (Fig. 3) by blocking CTLA-4, remodeling the immunosuppressive TME (Fig. 1 and Fig. 2) and normalizing tumor vasculature (Fig. 3). Whether or not the combination treatment could increase specific CTL killing activity. As shown in Fig. 4A, the PBS group and empty LCP group generated no significant MUC1-specific CTL response, a weak CTL response was observed in the naked mRNA-treated group, mice immunized with either MUC1 mRNA nanovaccine or anti-CTLA-4 mAb exhibited a moderate MUC1-specific CTL killing, and mice treated with combination therapy induced the strongest CTL response. IFN-γ is produced predominantly by cytotoxic T cells and plays a critical role in anti-tumor immunity. Therefore, IFN-γ is usually used as an alternative indicator of anti-tumor immune response [31]. ELISPOT assay was performed to measure IFN-γ production by lymphocytes. As shown in Fig. 4B, the groups immunized with either MUC1 nanovaccine or anti-CTLA-4 mAb stimulated modest IFN-γ secretion, and the highest IFN-γ production was observed in the combo group. Splenic cells isolated from mice in the naked mRNA group generated low level of IFN-γ. IFN-γ production was not observed in the PBS group and the empty LCP group. Immunosuppressive TME supports the rapid growth of tumor cells in mice inoculated with TNBC 4T1 cells. After mice were treated with anti-CTLA-4 mAb targeting CTLA-4, immunosuppressive TME was reduced and the infiltration of CD8^+^ T cells into the tumor was increased (Fig. 1 and Fig. 3), which facilitated to improve antigen-specific CTL killing and anti-tumor effect in mice receiving anti-CTLA-4 mAb (Supplementary Fig. S3). It has been reported that CTLA-4 siRNA-loaded nanoparticles could increase the number of CD8^+^ T cells, decrease the ratio of CD4^+^ FOXP3^+^ Tregs and effectively inhibited tumor growth in mice with melanoma [20].

### Expression of STAT3 and phospho-STAT3 in TME

**Fig. 5:**
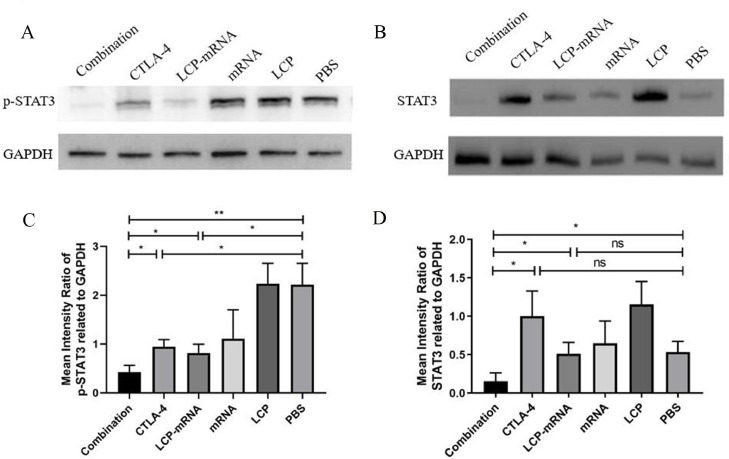
**Expression of STAT3 and phospho-STAT3 in TME.** (A, B) At the end of treatment (day 19), tumor tissues were harvested, and then the total tumor proteins were prepared. Expression of phospho-STAT3 and STAT3 in TME was detected by western blot assay. GAPDH was used as an internal control. (C, D) The band densities of proteins were quantified using Image J. The statistical analyses of the western blot results were shown. *p < 0.05, **p < 0.01, ns: no significant difference; *n* = 3.

STAT3 maintains a pro-carcinogenic inflammatory microenvironment during malignant transformation and cancer progression [32], and mediates the production of immunosuppressive cytokines in the TME, which is also a biomarker indicative of poor survival in TNBC [30, 33]. STAT3 is highly phosphorylated in many cell types in the TME, including Treg cells, myeloid-derived cells and tumor cells [30]. High levels of STAT3 are associated with unfavorable disease outcome and it was regarded as an important therapeutic target of TNBC [30, 33]. Therefore, the levels of STAT3 and phospho-STAT3 in tumor were examined by western blot assay. Fig. 5A-D showed that combined therapy greatly decrease the phospho-STAT3 and STAT3 levels in tumor, whereas monotherapy with either MUC1 mRNA nanovaccine or anti-CTLA-4 mAb exhibited only a modest reduction of phospho-STAT3. The STAT3 levels didn't significantly reduce in the nanovaccine group and the anti-CTLA-4 mAb group.

### TUNEL assay

**Fig. 6:**
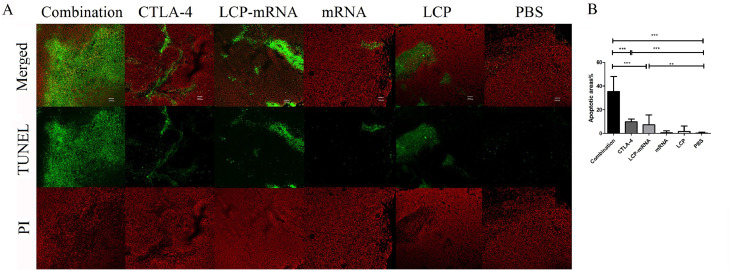
**Analysis of apoptosis in tumor by TUNEL assay.** (A) At the end of different treatments (day 19), tumor tissues were harvested. Apoptotic tumor cells in the paraffin-embedded tumor tissues were characterized by using TUNEL assay kit. The green represents the signal of the apoptosis and the red indicates nuclear staining by propidium iodide (PI). Bar=200 μm. (B). Quantitation of the TUNEL assay. Three selected randomly images were quantitatively analyzed using ImageJ software. The bar diagram shows the average count of triplicate results of TUNEL assay. **p < 0.01, ***p < 0.001.

Apoptosis in tumor sections can enhance the availability of tumor-associated antigens and initiate adaptive immune response [6]. Therefore, TUNEL assay was performed in tumor sections to evaluate tumor cell apoptosis. As shown in Fig. 6, MUC1 mRNA nanovaccine and anti-CTLA-4 mAb resulted in a significantly greater level of apoptosis in tumor cells when compared with PBS control group. In combination, MUC1 mRNA nanovaccine plus anti-CTLA-4 mAb appeared more effective than either nanovaccine or anti-CTLA-4 mAb alone at increasing level of apoptosis in tumor cells.

## Discussion

The immunosuppressive TME is the main reason for the failure of many immunotherapies that directly stimulate anti-tumor immune response [34]. High levels of CD4^+^T-regulatory cells in breast tumors are associated with reduced OS, whereas high levels of CD8^+^T cells combined with low levels of CD4^+^T-regulatory cells correlate with increased OS [35]. In other words, CD8^+^ T cells can control human breast cancer, but the presence of immunosuppressive cells reverses this action [35]. In this study, we examined the antitumor immune response of combined therapy with anti-CTLA-4 mAb and MUC1 mRNA nanovaccine and monitored the change of immunosuppressive immune cells and cytokines in the TME. The data demonstrated that combination treatment with anti-CTLA-4 mAb and MUC1 mRNA nanovaccine could reduce immunosuppressive TME, increase the infiltration of CD8^+^ T cells into tumor sites, and enhance anti-tumor cytotoxic T-lymphocyte activity when compared to monotherapy with either anti-CTLA-4 mAb or MUC1 mRNA nanovaccine.

Anti-CTLA-4 mAb therapy may enhance antitumor response by improving the antitumor function of CD8^+^ T cells, increasing the ratio of CD8^+^ T cells/Foxp3^+^ Tregs, and inhibiting the suppressive function of Tregs [35]. When CTLA-4 blockade is combined with melanoma cell vaccine, the ratio of effector T cells (Teff)/Treg induced in tumor is larger, which is directly associated with tumor rejection [36]. High ratio of Teff/Treg and Teff/MDSC are found in the tumor of melanoma-bearing mice treated with combination blockade of PD-1 and CTLA-4 [37]. The increase of Teff infiltration can abrogate the suppression of regulatory immune cells in tumor sites, so as to improve the effect of immunotherapy [38]. The combined treatment with MUC1 nanovaccine and CTLA-4 blocking antibody exhibited an obvious decrease in Treg and MDSC, a significant increase in the number of CD8^+^ T cells (Fig. 1), the strongest CTL response (Fig. 4) and a superior antitumor effect (Figure S3).

Signal transducer and activator of transcription 3 (STAT3) mediates tumor-induced immunosuppression at many levels [39]. Preclinical and clinical studies have shown that constitutive activation of STAT3 can be found in more than 50% of breast tumors, and most of it exists in TNBC [40]. TNBC is one of the most aggressive breast cancer subtypes, with poor prognosis and high metastatic capacity. The transcription factor STAT3 is a critical regulator of invasion and migration, and a biomarker indicative of poor survival in TNBC [33]. Many tumor-producing factors that are critical for tumor growth and immunosuppression, such as IL-6, IL-10 and VEGF, activate STAT3 to create an efficient “feedforward” mechanism to ensure increased STAT3 activity both in tumor cells and in tumor-associated immune cells [39]. Increased STAT3 activity promotes the accumulation of Tregs in the TME. STAT3 signaling in Tregs can upregulate the expression of TGF-β and IL-10, which conversely restrain IFN-γ production by CD8^+^ T cells, and finally inhibit the tumor-killing activity of CD8^+^ T cells [39]. In addition, immunosuppressive factors VEGF, IL-10 and IL-6 can be upregulated by STAT3, and these factors, in turn, inhibit the expression of immunostimulatory molecules, such as IL-12 and tumor-necrosis factor (TNF) [39]. Our results indicate that after combined treatment, the reduction of phosphorylated STAT3 activity results in a decrease of inhibitory cytokine IL-6 and TGF-β, and an increase of immunostimulatory molecule IL-12 and IFN-γ produced by CD8^+^ T cells, but TNF-α is reduced rather than increased. Actually, in the inflammatory tumor microenvironment of TNBC, TNF-α has been proved to be a major factor that triggers tumor cell immunosuppression against T-cell surveillance [2]. Therefore, after combined immunotherapy, inflammatory immunosuppressive tumor microenvironment was remodeled, the secretion of TNF-α should be reduced. It was reported that treatment of patients with cancer with PD-1 immune checkpoint inhibitor could induce the production of IL-6 [41]. Our data shown that monotherapy with anti-CTLA-4 mAb exhibited high level of IL-6, but the increase of CD8^+^ T cell infiltration and the reduction of Treg cells, TGF-β and phosphorylated STAT3 in the TME probably inhibited the immune suppression of IL-6 [38, 39]. The STAT3-dependent tumor-associates factors such as IL-10, VEGF and basic fibroblast growth factor (bFGF) could strengthen the immunosuppressive network and promote tumor vasculariztion that hinders IFN-γ-dependent effect of CD8^+^ T cells [39]. Thus, inhibiting STAT3 activity in tumor cells could reduce immunosuppression in the TME and enhance the effector function of CD8^+^ T cells.

Activation of STAT3 signaling pathway resulted in decreased apoptosis and promoted overall tumor growth [42]. Constitutive Stat3 activation early in head and neck carcinogenesis could enhance tumor progression by blocking tumor cell apoptosis [43]. Stat3 antisense gene therapy induced tumor cell apoptosis by targeting Stat3 [43]. Thus, STAT3 is considered as an important therapeutic target, and its inhibitors are currently being investigated in clinical trials for the treatment of TNBC [33].

In conclusion, combination treatment with anti-CTLA-4 mAb and MUC1 mRNA nanovaccine could increase the infiltration of CD8^+^ T cells into tumor sites and enhance anti-tumor cytotoxic T-lymphocyte activity by reducing immunosuppressive TME and inhibiting tumor-promoting STAT3 signaling pathway.

## Author contributions

The authors contributed in the following way: conception and design: Xuan Lin, Hedan Chen, Ying Xie, Zhu Zeng and Lina Liu; acquisition of data: Xuan Lin, Hedan Chen, Ying Xie, Xue Zhou, Yun Wang, Jing Zhou, Shiqi Long, Zuquan Hu, Shichao Zhang and Wei Qiu; writing-original draft: Xuan Lin, Hedan Chen and Ying Xie; writing-reviewing and editing: Zhu Zeng and Lina Liu.

## Declaration of Competing Interest

The authors declare no conflicts of interest for this work.

## Appendix Supplementary materials

Image, application 1
