# Supplementary material for: Combination of CTLA-4 blockade with MUC1 mRNA nanovaccine induces enhanced anti-tumor CTL activity by modulating tumor microenvironment of triple negative breast cancer
Source: Transl Oncol. 2021 Dec 4;15(1):101298. doi: 10.1016/j.tranon.2021.101298 (PMC8652013; doi:10.1016/j.tranon.2021.101298)
Supplement: Supplementary file 1 [file mmc1.docx]

**SUPPLEMENTAL INFORMATION for**

Combination of CTLA-4 Blockade with MUC1 mRNA Nanovaccine Induces Enhanced Anti-tumor CTL Activity by Modulating Tumor Microenvironment of Triple Negative Breast Cancer

Xuan Lin ^a, b, 1^, Hedan Chen ^a, 1^, Ying Xie ^a, 1^, Xue Zhou ^a^, Yun Wang ^a, c^, Jing Zhou ^a^, Shiqi Long ^c^, Zuquan Hu ^a^, Shichao Zhang ^a^, Wei Qiu ^a^, Zhu Zeng ^a, b, c,^ ^*^, Lina Liu ^a, b,^ ^*^

^a^ Key Laboratory of Biological and Medical Engineering/Immune Cells and Antibody Engineering Research Center of Guizhou Province/Engineering Research Center of Medical Biotechnology, School of Biology and Engineering, Guizhou Medical University, Guiyang, Guizhou 550025, PR China;

^b^ Key Laboratory of Environmental Pollution Monitoring and Disease Control, Ministry of Education, Guizhou Medical University, Guiyang, Guizhou 550025, PR China;

^c^ School of Basic Medical Science, Guizhou Medical University, Guiyang, Guizhou 550025, PR China

***Corresponding authors**

Lina Liu, Key Laboratory of Biological and Medical Engineering, School of Biology and Engineering, Guizhou Medical University, Guiyang, Guizhou 550025, PR China. **E-mail**: [strawberry2@126.com](mailto:strawberry2@126.com);

Zhu Zeng, Key Laboratory of Biological and Medical Engineering, School of Biology and Engineering, Guizhou Medical University, Guiyang, Guizhou 550025, PR China. **E-mail**: [zengzhu@gmc.edu.cn](mailto:zengzhu@gmc.edu.cn)

^1^ These authors contributed equally to this work.

***Keywords****:* MUC1 mRNA nanovaccine, CTLA-4 blockade, Combined therapy, Tumor microenvironment, Triple negative breast cancer

**Supplemental Figures**

**Fig. S1. *In vitro* transcription and expression of MUC1 mRNA.** (A) Agarose gel electrophoresis of *in vitro* transcribed MUC1 mRNA. (B) Transient expression of *in vitro* transcriptionally modified MUC1 mRNA in mammalian 4T1 cells detected by western blot assay.


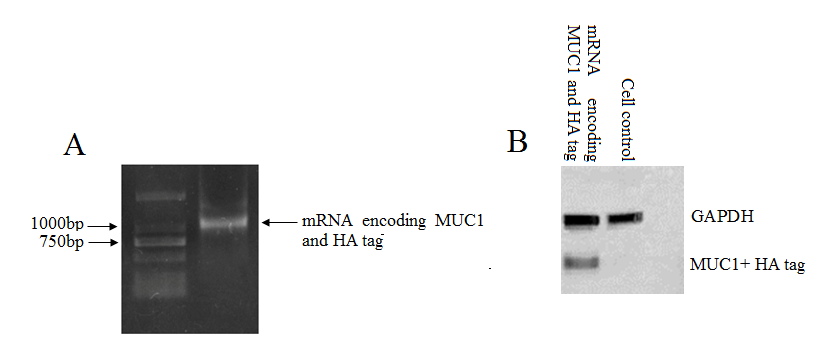


**Fig. S2. The TEM image of nanoparticle.** (A) TEM images of CaP cores. (B) TEM images of LCP NPs after negative staining.


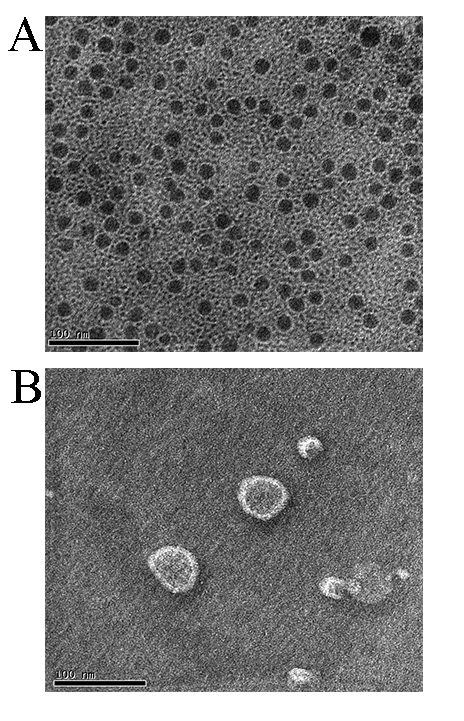

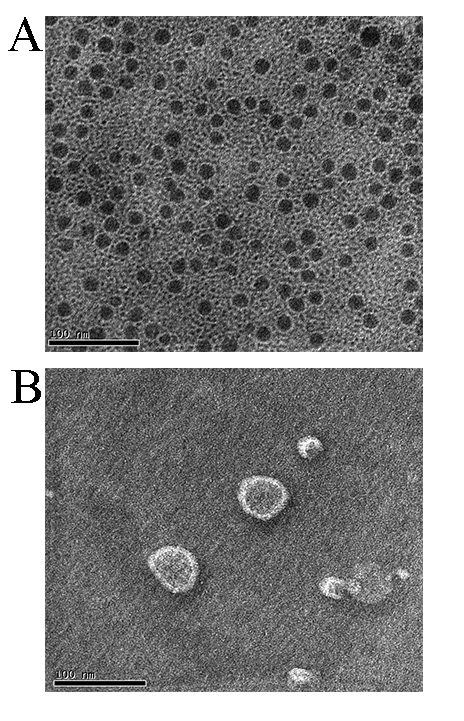

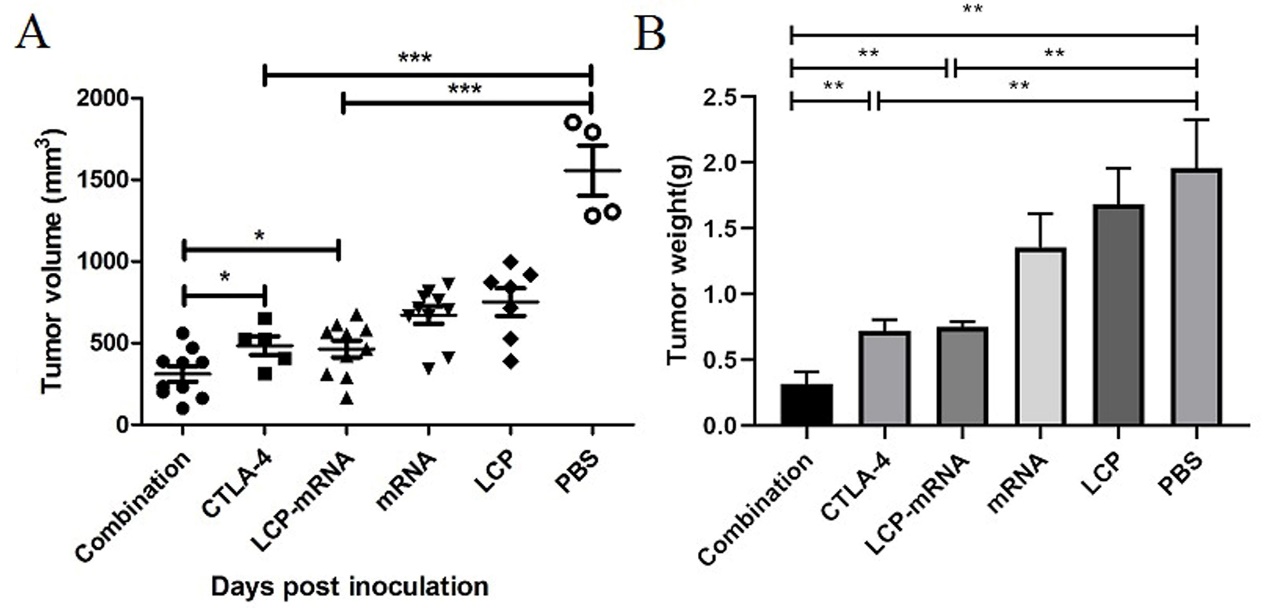


**Fig. S3. Tumor growth inhibition experiment.** (A) Analysis of tumor volume at the end of treatment (day 19). Bars represent standard error of mean. Statistical significance was analyzed using a two-tailed t test. (B) Tumor weight at the end of experiment (day 19). *p < 0.05, **p < 0.01, ***p < 0.001.
